# A combination of faecal and intratumour microbial community profiling reveals novel diagnostic and prognostic biomarkers for pancreatic tumours

**DOI:** 10.1002/ctm2.1726

**Published:** 2024-05-31

**Authors:** Wei Wang, Cheng Qian, Ting Wang, Yuetong Jiang, Yiran Zhou, Kaiyu Liu, Zhiyang Ma, Pengyi Liu, Yichi Wu, Leying Chen, Huaizhi Wang, Tingting Zhou

**Affiliations:** ^1^ Department of Gastroenterology Shanghai General Hospital Shanghai Jiao Tong University School of Medicine Shanghai China; ^2^ National Key Laboratory of Immunity & Inflammation Institute of Immunology Second Military Medical University Shanghai P. R. China; ^3^ Department of Pathology Ruijin Hospital, School of Medicine Shanghai Jiao Tong University Shanghai P. R. China; ^4^ Shanghai Key Laboratory for Pharmaceutical Metabolite Research School of Pharmacy Second Military Medical University Shanghai P. R. China; ^5^ Department of General Surgery and Research Institute of Pancreatic Diseases Ruijin Hospital, School of Medicine Shanghai Jiao Tong University Shanghai P. R. China; ^6^ School of Medicine Shanghai Jiao Tong University Shanghai P. R. China; ^7^ Department of Liver Surgery, Renji Hospital School of Medicine Shanghai Jiao Tong University Shanghai P. R. China; ^8^ Institute of Hepatopancreatobiliary Surgery Chongqing General Hospital Chongqing University Chongqing P. R. China

Dear Editor,

Our findings provide a novel interpretive framework for diagnostic and prognostic biomarkers for pancreatic tumours. A multi‐step combination of faecal and intratumour microbial classifiers has the potential to be clinical biomarkers in the early detection of pancreatic cancer, which has less invasiveness, higher sensitivity and specificity.

Pancreatic tumours are extremely lethal diseases characterized by difficult early diagnosis and rapid metastasis.^1^ Histopathological examination of the biopsy specimens of pancreatic tumours obtained by endoscopy or surgery is considered as a gold diagnosis standard of pancreatic tumors.^2^ Nonetheless, pathological analysis requires very high accuracy in biopsy localization with multiple sampling, and the accuracy of sampling has a significant impact on misjudgment.^3^ Given diagnostic delays, cancer metastasis and adverse reactions of chemotherapy or radiotherapy, it is urgent to explore new strategies which are required to develop effective diagnostic and treatment options for improving clinical outcomes of pancreatic tumours.

As a novel component in shaping the immune system, the microbiome has been increasingly shown to be important in the maintenance of homeostasis in several physiologic processes.^4^ Here, we used 193 faecal samples (156 patients with different grade of human pancreatic tumours and 37 healthy volunteers) and 362 fresh pancreatic tissues of malignant (PDAC), premalignant (IPMN1 and IPMN2) and benign (SCN) patients from different hospitals (training and validation cohorts) to identify the link between the abundance and diversity changes of faecal and intra‐pancreatic microbes and the prognosis and prognostic of pancreatic tumours. The basic statistical conditions are as follows: the difference between the study group and the healthy control group is tested with a confidence level of 80% and a ratio of .1000 between groups. The study group is assumed to be .05 and .15 under the null hypothesis and alternative hypothesis, respectively, while the healthy control group is assumed to be 0.05. Use the method of bilateral Z‐test and combined variance to test the statistics. The statistical difference was set to .05. Based on previous foreign data and research on gut microbiota in adolescents with idiopathic chronic pancreatitis, two lowest and most conservative data, a positive rate of 30% in the study group and 10% in the healthy control group, were used for estimation. The required sample size for the study group (PDAC) was 59 cases. Considering factors such as errors, the final number of cases was determined to be 90.

## 1 STAGE‐SPECIFIC GUT MICROBIAL SIGNATURES ARE DIFFERENTIALLY RICHED AS NON‐INVASIVE BIOMARKERS FOR EARLY DIAGNOSIS OF PANCREATIC TUMORS

**FIGURE 1:**
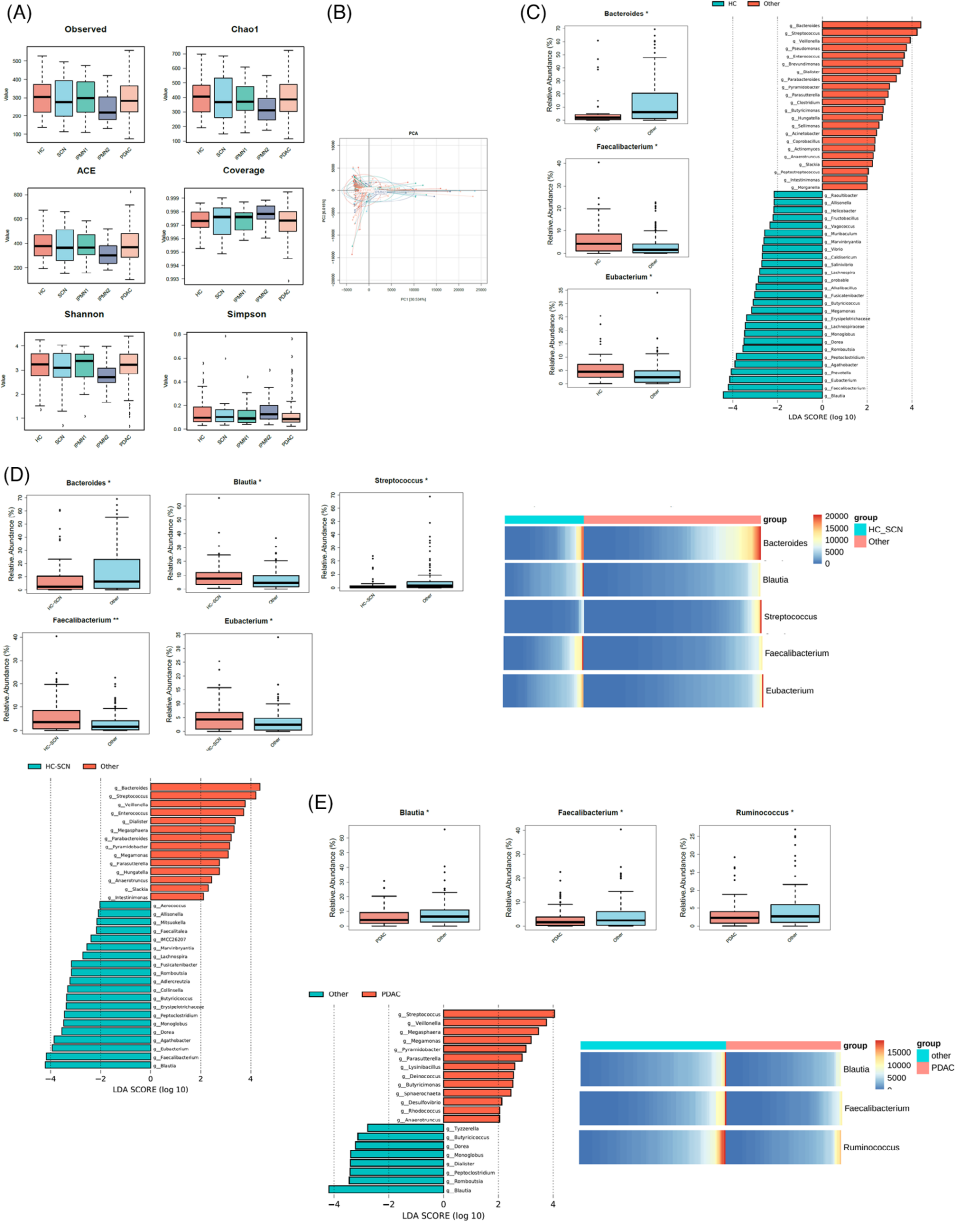
Faecal microbial characteristics represented potential noninvasive tools for early screening of human pancreatic tumours. (A) Alpha diversity boxplot (Observed species, Chao1, ACE, Coverage, Shannon and Simpson reciprocal) in all stool samples from healthy controls (HC) and different grade of human pancreatic tumours, including SCN, IPMN1, IPMN2 and PDAC patients. (SCN, serous cystic neoplasm; IPMN1, intraductal papillary mucinous neoplasm of the pancreas with low‐grade dysplasia; IPMN2, intraductal papillary mucinous neoplasm of the pancreas with high‐grade dysplasia and associated invasive carcinoma; PDAC, pancreatic ductal adenocarcinoma). (B) Principal component analysis (PCA) of the faecal microbiome composition based on weighted UniFrac distance from different grade of human pancreatic tumors and healthy controls on the operational taxonomic unit (OTU) level. The shapes and colours of the points indicate samplesfrom each patient. (C) Taxonomic distribution of gut microbiota between HC and SCN, IPMN1, IPMN2 and PDAC cohorts is shown. Relative abundance of the remaining phyla expressed as a percentage of the biomass of the “Other” phyla from SCN, IPMN1, IPMN2 and PDAC cohorts. (*p < 0.05). Linear discriminant analysis (LDA) analysis of effect size (LEfSe) was used to determine the differentially enriched genera in stools between HC and the “Other” cohorts from SCN, IPMN1, IPMN2 and PDAC patients. LDA data computed from features differentially abundant between healthy controls and “Other.” The criteria for feature selection are log LDA score > 4. D: Taxonomic distribution of gut microbiota between HC and SCN cohorts (HC‐SCN) and IPMN1, IPMN2 and PDAC cohorts (Other) is shown. Relative abundance of the remaining phyla expressed as a percentage of the biomass of the “Other” phyla from IPMN1, IPMN2 and PDAC cohorts. (*p < 0.05). LDA analysis was used to determine the differentially enriched genera in stools between HC‐SCN and “Other.” The criteria for feature selection are log LDA score > 4. The heatmap presents the differentially enriched genera in stools between HC‐SCN and “Other.” (E) Taxonomic distribution of gut microbiota between PDAC patients and HC, SCN, IPMN1 and IPMN2 cohorts (Other) is shown. Relative abundance of the remaining phyla expressed as a percentage of the biomass of the “Other” phyla from HC, SCN, IPMN1 and IPMN2 cohorts. (*p < 0.05). LDA analysis was used to determine the differentially enriched genera in stools between PDAC patients and “Other.” The criteria for feature selection are log LDA score >4. The heatmap presents the differentially enriched genera in stools between PDAC controls and “Other.”

At the phylum level, faecal samples from different grade of pancreatic lesion and healthy control groups are relatively close, indicating that the sample composition is similar (Figure 1A, B). To further investigate these findings, we contrasted the microbial genera between healthy controls and pancreatic tumour patients including SCN, IPMN1, IPMN2 and PDAC. Interestingly, linear discriminant analysis (LDA) revealed that the abundance of specific bacterial genera in stools, including *Faecalibacterium*, *Eubacterium*, *Bacteroides*, *Blautia*, *Streptococcus* and *Ruminococcus*, may be used as non‐invasive biomarkers to early diagnose the multistep progression of pancreatic tumours (Figure 1C–E).

## 2 HUMAN INTRA‐PANCREATIC MICROBIOME PROFILING IS ASSOCIATED WITH INCREASING GRADE OF MALIGNANCY OF PANCREATIC TUMORS

**FIGURE 2:**
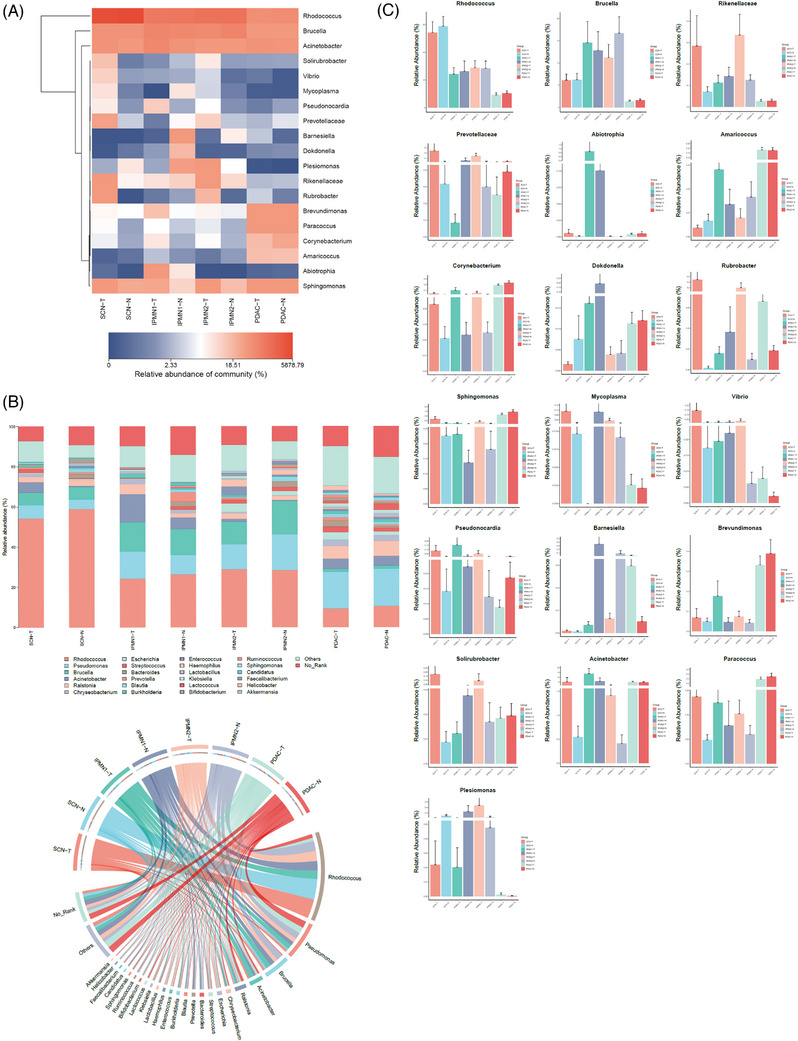
The different grade of human pancreatic tumours harbours distinct intra‐pancreatic microbiomes. (A) Heatmap of the top 19 genera in the bacterial communities in the pancreatic tissues from SCN, IPMN1, IPMN2 and PDAC cohorts including related tumour‐adjacent normal tissue (NAT) cohorts. The relative abundance of bacterial genera was normalized as indicated by the colour gradient. Double hierarchical linkage clustering of the samples was based on microbial composition and abundance. (B) Distribution of genus level across different pancreatic tumour types. Relative abundances were calculated by summing up the reads of species that passed all filters in the different tissue types and belong to the same genus. Circos plots showing correlations between genus abundances interacting with SCN, IPMN1, IPMN2 and PDAC cohorts. The down side lists the name of the main pancreatic microbial genera. The up‐side of circle shows SCN, IPMN1, IPMN2 and PDAC cohorts (SCN‐T, IPMN1‐T, IPMN2‐T and PDAC‐T) with their NAT cohorts (SCN‐N, IPMN1‐N, IPMN2‐N and PDAC‐N). The inner lines of the up‐side of circle show distribution of genera across different pancreatic lesions types. Each ribbon represent genus‐phenotype associations indicates a significant association between a microbial factor and different pancreatic tumour types with line colour corresponding to the associated microbial genera. (C) Taxonomic distribution of the top 19 genera in the bacterial communities in the pancreas of different pancreatic tumour types and their NATs cohorts is shown.

To determine the differences of the microbiome composition among SCN, IPMN1, IPMN2 and PDAC pancreatic tumours, we interrogated the bacterial profiles of fresh pancreatic tissues samples from PDAC and various non‐malignant pancreatic tumour patients. Although groups did not separate on PCA based on their microbiomes, similar to the observations that are derived from alpha‐diversity index data, PDAC and the related NAT groups showed the greatest separation from the other groups (Figure S1). Considering the potential connection between pancreatic microbiome diversity and malignant degree of pancreatic tumours, we next need to determine the differences of the pancreas microbial composition between non‐malignant pancreatic tumours and PDAC. Notably, the distribution of genus‐level revealed marked changes in the bacterial composition of the different pancreatic tumour types (Figure 2A). Bacteria belonging to the *Pseudomonas* and *Ralstonia* genera were the most abundant species in PDAC and their NATs. In contrast, *Rhodococcus* and *Brucella* were more abundant in the non‐malignant pancreas (IPMN1, IPMN2, SCN) and their related NAT groups. We also detected the proportions of significantly increased genera in different pancreatic tumour types (Figure 2B). *Pseudomonas* was the dominant genus, but no significant differences were observed among different cohorts. However, *Rhodococcus* was abruptly enriched in the nonmalignant pancreas cohorts, whereas *Brucella* was more abundant in SCN and their NAT. Although *Acinetobacter* was present in low abundance in the pancreas, it increased obvious abundance in the IPMN1 cohorts (Figure 2C).

## 3 EVALUATION AND VALIDATION OF PDAC‐ASSOCIATED MICROBIOTA DIAGNOSTIC BIOMARKERS

**FIGURE 3:**
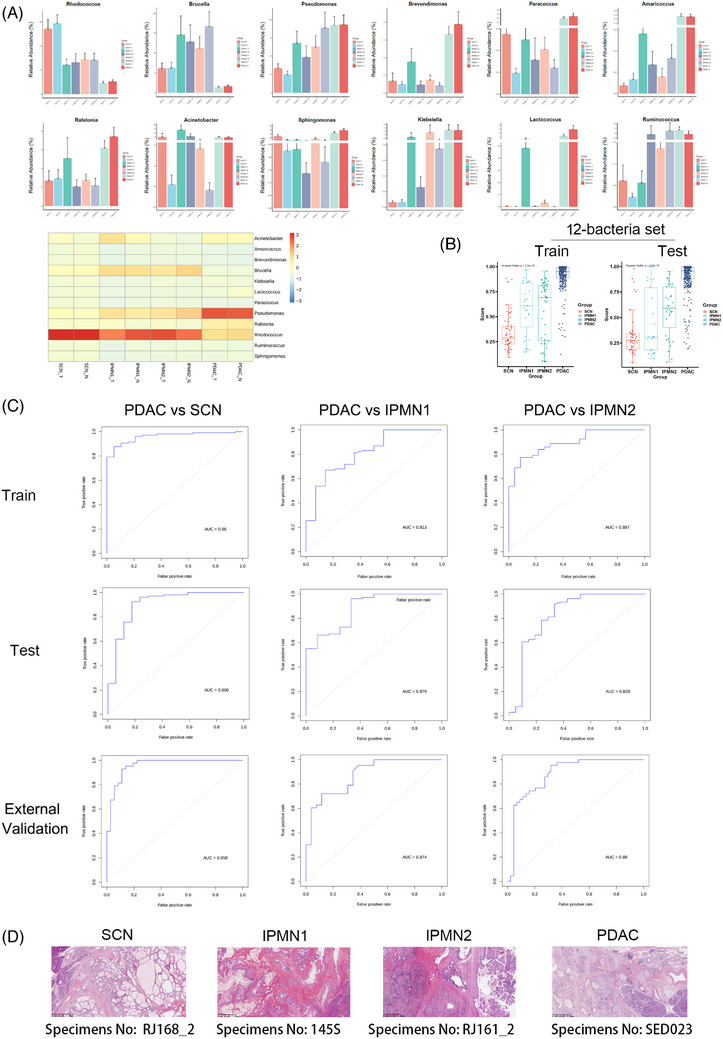
The 12‑bacteria biomarkers had a favourable performance in distinguishing PDAC and other pancreatic tumour tissues and can be used as diagnostic biomarkers of the degree of malignancy of pancreatic tumours. (A) Plots of the 12 differentially abundant bacteria significantly enriched in the pancreatic tissues from SCN, IPMN1, IPMN2 and PDAC cohorts including related NAT cohorts. Heatmap demonstrated the differentially enriched genera in the pancreatic tissues across the different tumour types. (B) A significant difference of the risk score of the 12‐bacteria set was shown to separate SCN, IPMN1, IPMN2 and PDAC cohorts from each other. The multi‑bacteria score models were trained in aggregate in the risk score of discovery cohort (left panel) and then were tested in the validation cohort (right panel). Each dot represents a sample. (C) ROC curves analysis to evaluate the discriminatory potential of risk score of the 12‐bacteriaset in PDAC versus SCN, PDAC versus IPMN1and PDAC versus IPMN2 detection in the discovery cohort (up panel) and then were tested in the validation cohort (middle panel). External validation showed that optimized model was better and stable in diagnosing PDAC (down panel).(D) Histologic analysis of SCN (Specimens No: RJ168_2), IPMN1(Specimens No:145S), IPMN2 (Specimens No: RJ161_2) and PDAC (Specimens No: SED023) on paraffin sections. Four representative H&E specimens from PDAC versus SCN, PDAC versus IPMN1 and PDAC versus IPMN1 in the validation cohorts, with low or high risk score of 12‐bacteriaset, respectively, are shown.

We used the patient's risk score as a variable to observe whether the abundance of OUTs at the genus level can segregate the intra‐tumour microbiome (Figure 3A). Next, we attempted to assess the mean relative abundance differences of species from both benign and premalignant to malignant pancreatic tumours which might increase potential value of diagnosing the grades of malignancy. We visualized a significant differential segregation between the communities of PDAC and non‐malignant pancreatic tumours according to the risk scores, and found PDAC was associated with a higher‐risk value than the control groups (Figure 3B and Figure S2). The 12‐bacteria signature (*Rhodococcus*, *Brucella*, *Pseudomonas*, *Kiebsiella*, *Lactococcus*, *Ruminococcus*, *Brevundimonas*, *Paracoccus*, *Amaricoccus*, *Ralstonia*, *Acinetobacter and Sphingomonas*) had a more favourable performance in the trained cohorts, which then validated in the tested cohorts. The results were further confirmed by the external validation study including another centre to determine diagnostic biomarkers (Figure 3C). We next tested pancreatic tissues to confirm our findings obtained in intra‐tumour microbiom by histologic analysis. These results all indicated that these twelve genera with different abundances had an obvious effect in distinguishing PDAC and malignant pancreatic tissues and could be used as diagnostic biomarkers with a certain degree of accuracy.

## 4 INTRA‐PANCREATIC MICROBIAL DIVERSITY INFLUENCES THE SURVIVAL OF PDAC PATIENTS

To explore the relationship between the human intra‐pancreatic microbiome composition and the survival rate of PDAC patients, we carried out Kaplan–Meier survival analysis and machine learning analysis. The result suggested that the survivorship of PDAC patients could be influenced and predicted by the abundance of 11 taxonomic communities, including *Blautia*, *Faecalibacterium*, *Comamonas*, *Coprococcus*, *Erysipelatoclostridium*, *Sulfuritalea*, *Thermomonas*, *Phenylobacterium*, *Raoultibacter*, *Erysipelothrix*, *Thiothrix* (Figure S3).

## 5 DIFFERENT METABOLIC PATHWAYS DRIVED BY MICROBIOME COMMUNITIES FROM THE DIFFERENT GRADES OF HUMAN PANCREATIC TUMORS

**FIGURE 4:**
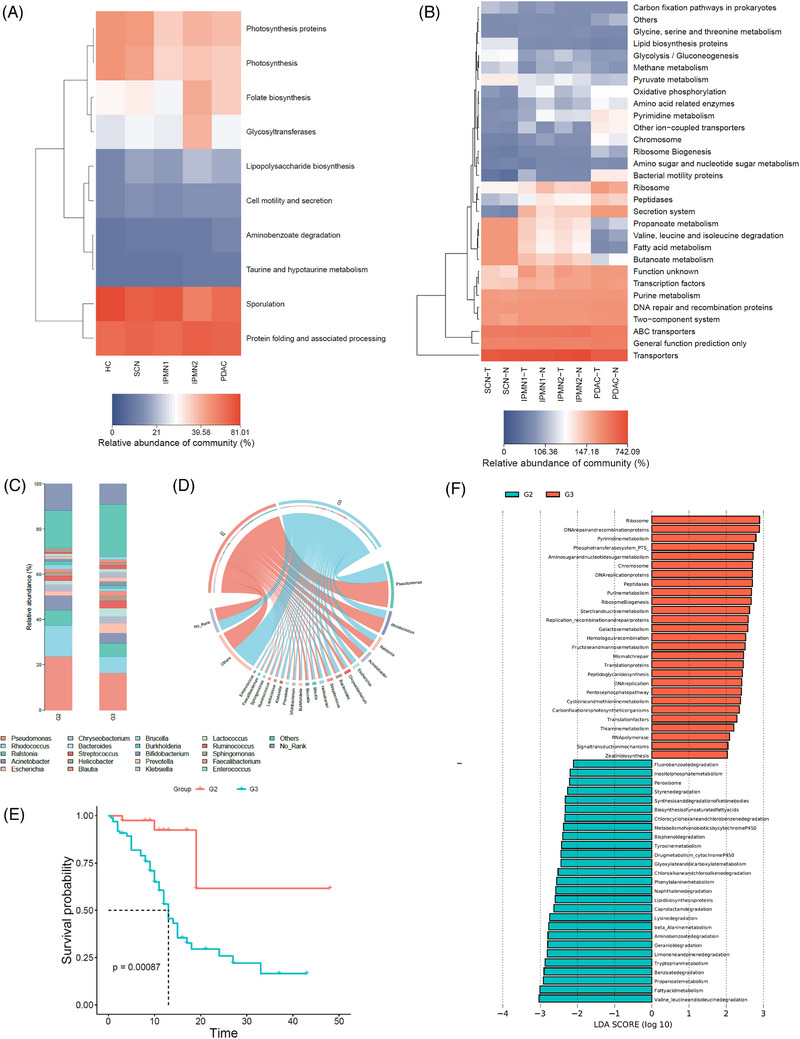
Microbiome communities from the different grade of human pancreatic tumours drive different metabolic pathways. (A) A heatmap of PICRUSt analysis identified ten core metabolic functional modules present across the stool samples from HC and different grade of human pancreatic tumour, including SCN, IPMN1, IPMN2 and PDAC patients. (B) A heatmap of PICRUSt analysis identified 30 core metabolic functional modules present across the pancreatic tissues from SCN, IPMN1, IPMN2 and PDAC cohorts including related NAT cohorts. (C) The relative abundances of major genera in PDAC G2 (pancreatic ductal adenocarcinomas with tumour grade 2) and PDAC G3 (pancreatic ductal adenocarcinomas with tumour grade 3) patients. (D) Circosplots showing down‐side of circle (the main pancreatic microbial genera) and up‐side of circle (PDAC G2 and G3 patients) for the changes in correlational links between the pancreatic microbiome at the genus level and PDAC G2 and G3 cohorts. The inner lines of the up‐side of circle show distribution of genera across PDAC G2 and G3 cohorts. Each line indicates a significant association between a microbial factor and PDAC G2 and G3 cohorts with line colour corresponding to the associated microbial genera. (E) Kaplan‐Meier estimates for survival probability based on the enrich metabolic pathways in PDAC G2 and G3 patients. (*p* < 0.001). (F) LDA score computed from enrichment metabolic pathways between PDAC G2 and G3 patients.

There is evidence to suggest that changes in the composition of the gut microbiome are a characteristic of metabolic disorders, meanwhile the metabolic syndrome has a significant impact on our health and could increase the probability of developing cancer.^5^, ^6^, ^7^ To assess if the faecal and pancreatic microbiota have relationship with the metabolic pathways, reference genome databases were used to predict metagenomes through PICRUSt analysis (Phylogenetic Investigation of Communities by Reconstruction of Unobserved States). Ten core functional modules were identified in faecal samples, meanwhile 30 core functional modules were identified in tissue samples of SCN, IPMN1, IPMN2 and PDAC cohorts including related NAT cohorts (Figure 4A,B). Twenty distinct intrapancreatic phyla were detected in PDAC Grade2 and Grade3, and the potential interactions were predicted between them, suggesting that the distinct phyla may support PDAC progression through various metabolic mechanisms(Figure 4C–F).

In conclusion, composite microbial communities may serve as diagnostic and prognostic biomarkers for pancreatic tumours in the early stage, and this strategy is a promising approach with less invasiveness, higher sensitivity and specificity.

## AUTHOR CONTRIBUTIORS

Wei Wang, Cheng Qian, Ting Wang, Yuetong Jiang, Yiran Zhou, Kaiyu Liu, Zhiyang Ma, Pengyi Liu, Yichi Wu, Leying Chen, Huaizhi Wang and Tingting Zhou conducted the experiment and analysed the data. Wei Wang carried out experiments. Cheng Qian wrote the initial manuscript. Ting Wang analysed the data. Yuetong Jiang helped revised the manuscript and designed the graphical abstract. Yiran Zhou, Kaiyu Liu, and Zhiyang Ma helped analyse 16S rRNA sequencing data. Pengyi Liu, Yichi Wu, Leying Chen helped to provide clinical sample and performed data analyses. Tingting Zhou conceived and initiated the project. Wei Wang, Tingting Zhou and Huaizhi Wang revised the manuscript. Tingting Zhou and Cheng Qian directed the project. Wei Wang was responsible for the drafts of the manuscript, as well as all requested revisions.

## CONFLICT OF INTEREST STATEMENT

The authors declared no competing interests.

## ETHICS STATEMENT

This study was approved by the Institutional Review Board (IRB) of Shanghai Jiao Tong University, and the protocols were approved by the Committee of Human Subjects Protection of Ruijin Hospital.

## CONSENT TO PARTICIPATE

Informed consent was obtained from the parents of all recruited children. The clinical trial registry number is NCT03809247 (https://register.clinicaltrials.gov/).

## Supporting information

Supporting Information

## Data Availability

All data are present in the main text. The data that support the findings of this study are openly available in NCBI SRA at https://www.ncbi.nlm.nih.gov/bioproject/?term=PRJNA830331. The accession number for the data is PRJNA830331.
