# Supplementary material for: A combination of faecal and intratumour microbial community profiling reveals novel diagnostic and prognostic biomarkers for pancreatic tumours
Source: Clin Transl Med. 2024 May 31;14(6):e1726. doi: 10.1002/ctm2.1726 (PMC11142927; doi:10.1002/ctm2.1726)
Supplement: Supplementary file 1 — Supporting Information [file CTM2-14-e1726-s001.docx]

**
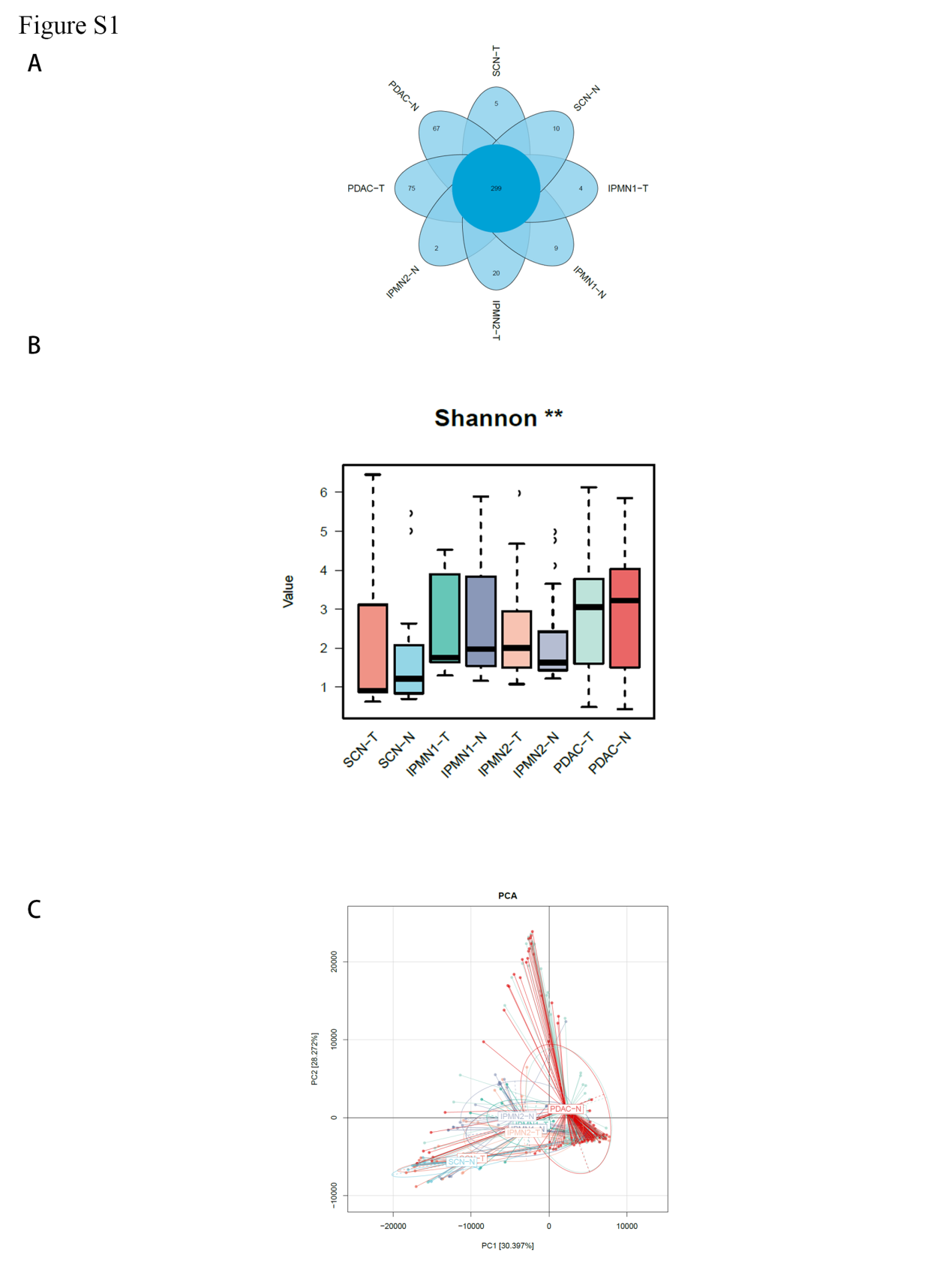
Figure S1. [Intra-pancreatic](#_Hlk67582966" \s "1,0,16,0,,Intra-pancreatic)microbial diversity influences the degree of malignancy of pancreatic diseases.**

A: Flower plots showing numbers of species-specific bacteria commonly found in fresh tissue samples fromeach histologic type of pancreatic tumors including related NATs (in the petals), and core bacteria number of pancreatic tumors (in the center).

B: The pancreatic microbiome of different grade of human pancreatic lesions were tested for differences in alpha-diversity indice of Shannon. (**p<0.01).

C: Relationships between microbial communities for the pancreatic samples from SCN, IPMN1, IPMN2 and PDAC cohorts including related NAT cohorts were analyzed by PCA based on weighted UniFrac distance. The data indicate eight distinct clusters representing each cohort. Variations are shown on the x- and y-axes. The shapes and colours of the points indicate samples from each patient. The coloured ellipses indicate 0.95 confidence interval (CI) ranges within each tested group.


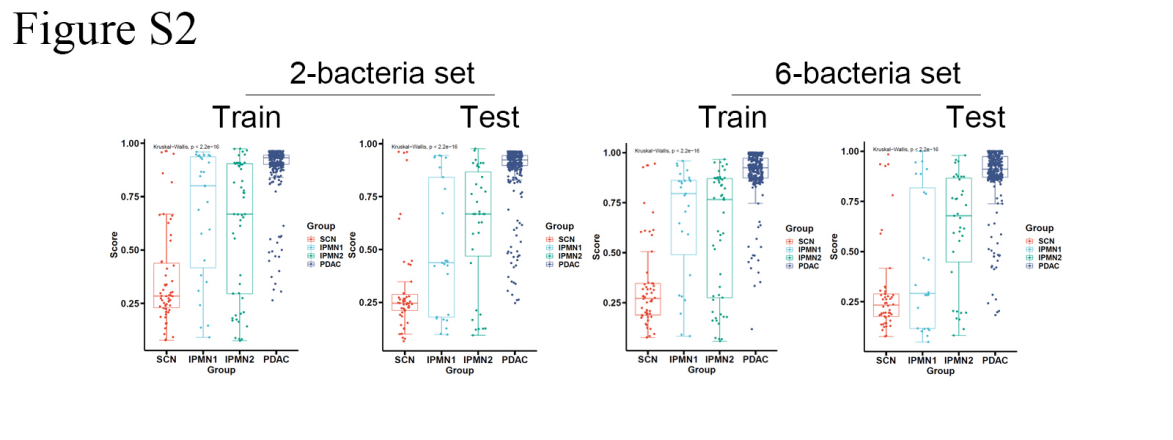


Figure S2: A significant difference of the risk score of the 2-bacteria set or 6-bacteria set was shown to separate SCN, IPMN1, IPMN2 and PDAC cohorts from each other. The multi‑bacteria score models were trained in aggregate in the risk score of discovery cohort (left panel) and then were tested in the validation cohort (right panel). Each dot represents a sample.

**
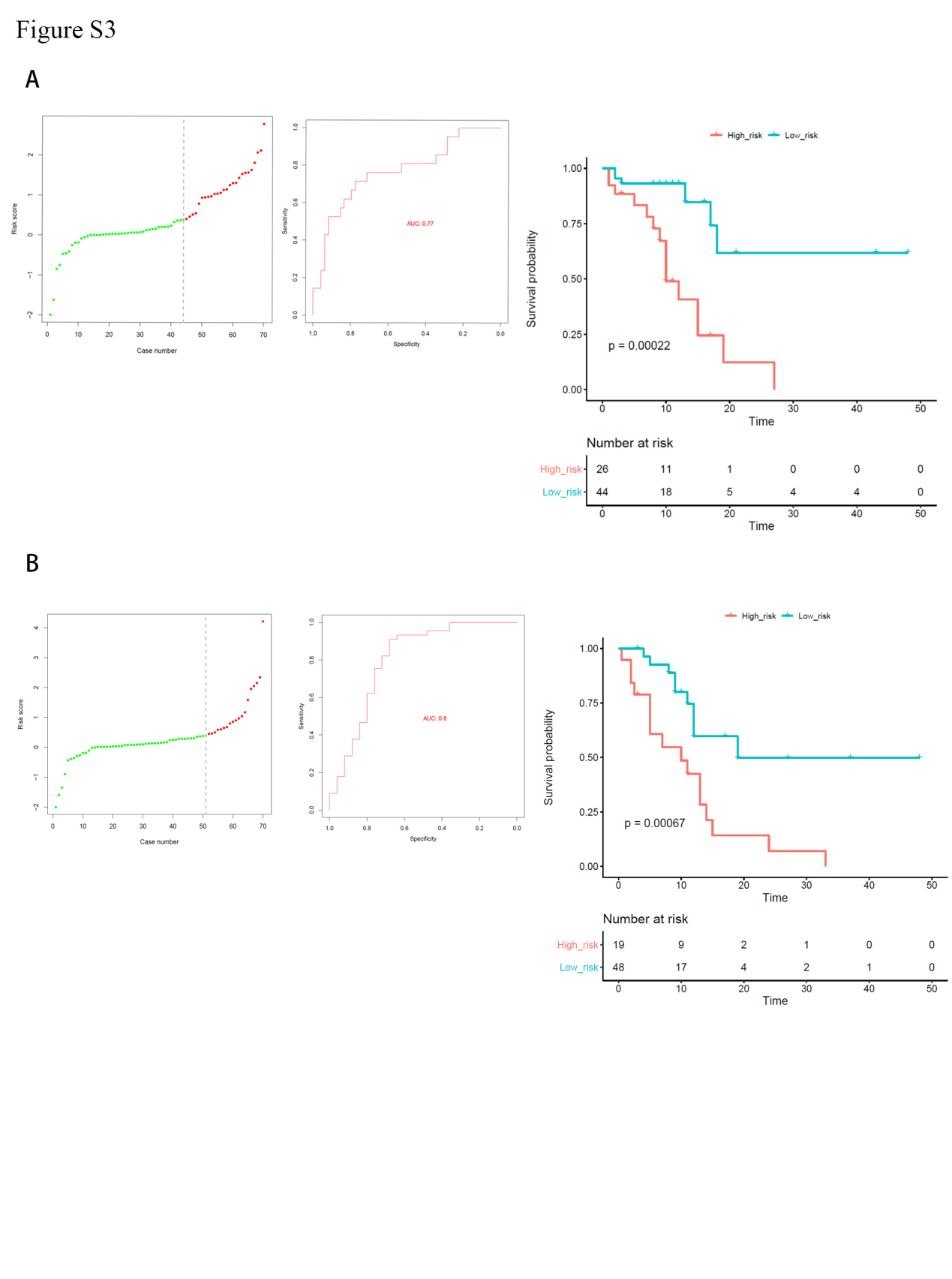
Figure S3. Prediction of survival of PDAC patients based on tumor microbial diversity -based classification models.**

A. Y-axis represents the probability of samples predicted as “high-risk.” X-axis represents ordered samples. Each dot represents a sample (red is high-risk, green is low-risk).ROC curves analysis to evaluate the discriminatory potential of 11-genera signature in the detection of high-risk PDAC patients in the discovery cohort. Kaplan-Meier survival curves of overall survival (OS) among pancreatic cancer patients from high-risk groups and low-risk group stratified by the signature in the training set.

B. The same algorithm was performed for further examination in the validation set.
